# Role of Cone-Beam Computed Tomography (CBCT) in Obstructive Sleep Apnea (OSA): A Comprehensive Review

**DOI:** 10.3390/diagnostics16020298

**Published:** 2026-01-16

**Authors:** Maudina Dwi Heriasti, Firdaus Hariri, Hui Wen Tay

**Affiliations:** 1 Department of Oral and Maxillofacial Clinical Sciences, Faculty of Dentistry, University of Malaya, Kuala Lumpur 50603, Malaysia; maudinaheriasti@gmail.com (M.D.H.); firdaushariri@um.edu.my (F.H.); 2 Oral & Maxillofacial Surgery Department, Cipto Mangunkusumo Hospital, Jakarta 10430, Indonesia; 3 Hospital Sultanah Aminah Johor Bahru, Jalan Persiaran Abu Bakar Sultan, Johor Bahru 80100, Malaysia

**Keywords:** airway, cone-beam computed tomography CBCT, obstructive sleep apnea

## Abstract

Obstructive sleep apnea (OSA) is characterized by recurrent partial or complete upper airway collapse during sleep. Accurate assessment of airway anatomy is crucial for risk stratification, diagnosis, and treatment planning. While polysomnography (PSG) is considered the gold standard for OSA diagnosis, it provides limited anatomical insights. Cone-beam computed tomography (CBCT) has emerged as a valuable tool with lower radiation dose for three-dimensional (3D) assessment of the upper airway space and craniofacial structures. CBCT enables precise measurement of critical airway parameters including total airway volume and length, minimum cross-sectional area, linear dimensions of anteroposterior and lateral diameters, as well as soft tissue structures such as tongue, tonsils, and adenoids. This review aims to explore and comprehensively review the role of CBCT, primarily in upper airway assessment for OSA, with an emphasis on airway measurement parameters, anatomical reference landmarks, and the variabilities, in addition to its clinical applications in treatment planning and simulation and post-treatment efficacy evaluation. This review also highlights the technical considerations such image acquisition protocols, machine specifications and software algorithm, and patient positioning, which may affect measurement reliability and diagnostic accuracy. CBCT serves as a powerful adjunct in OSA diagnosis and management, enabling comprehensive assessment of the airway space and hard and soft tissue structures. It complements PSG by guiding personalized interventions such as maxillomandibular advancement or CPAP optimization. Standardized imaging protocols and consideration of patient positioning can further improve its clinical utility.

## 1. Introduction

Obstructive sleep apnea (OSA) is a prevalent sleep-breathing disturbance that involves total or partial upper respiratory tract collapse during sleep. This condition is associated with cardiovascular, metabolic, and neurological conditions, and significantly impacts the overall quality of life and health outcomes. Several diagnostic methods are available to identify OSA, including polysomnographic (PSG) as the gold standard. PSG identifies the presence and severity of OSA but lacks structural insights on the structural causes of airway obstruction. As such, imaging modalities are increasingly employed to complement PSG findings. Traditionally, lateral cephalogram has served as a crucial tool for evaluating craniofacial skeletal patterns and posterior airway space in OSA patients. However, two-dimensional imaging may not be able to deliver the complex anatomical structure of the craniofacial region and airway [1,2,3]. In recent years, cone-beam computed tomography (CBCT) has emerged as a useful diagnostic imaging for assessing upper airway obstruction in OSA patients. CBCT provides a comprehensive assessment and three-dimensional analysis of craniofacial features [4,5]. CBCT shows the complex structure of the airway, which cannot be captured by a conventional lateral cephalogram. It also has a relatively low dose of radiation and shorter scan time compared to a computed tomography (CT) scan [3].

Over the past decade, CBCT has been increasingly adopted as a reliable tool for upper airway measurement for diagnostic, treatment planning, analysis, or post-treatment evaluation. CBCT can provide accurate evaluations of upper airway volume, length, width, and shape from various planes (axial, sagittal, coronal) [3,6]. Furthermore, 3D reconstruction can be provided to allow visualization of anatomical reference landmarks for craniofacial volumetric structures [7]. In pediatric patients, CBCT is especially useful to assess soft tissue obstruction such as hypertrophic adenoids or tonsils in the upper airways, which may attribute to OSA [8].

Anatomic landmarks can be identified correctly through CBCT using a combination of anatomical planes and 3D reconstruction of the skull. CBCT can obtain more accurate evaluations of (1) total airway volume, (2) narrowest cross-sectional area of the posterior airway space [6,9], (3) narrowest width from axial view according to the anteroposterior (AP) and lateral (L) dimension, and (4) airway length. CBCT also provides 3D reconstruction to allow visualization of anatomical reference landmarks for craniofacial volumetric structures [3,7].

Studies have highlighted the importance of postural effects which should be taken into account when assessing upper airway structure, especially in OSA patients, as airway dimensions can vary significantly between upright and supine positions. Ideally, CBCT should be performed under a supine position to show more predictive upper airway obstruction result, where OSA typically manifests during sleep [8]. However, a study showed that there is no association between head posture and pharyngeal airway, thus some studies used an upright position for CBCT acquisition approach [7].

CBCT provides detailed evaluation of the three-dimensional analysis from the craniofacial structure, and shows high contrast between bone, empty spaces, and soft tissue in general [3]. Accurate measurement of CBCT imaging shows high reliability compared to CT for upper airway measurement. However, limitations on assessing the posterior airway space arises from the dynamic space and constantly changing volume, area, and shape, which are dependent on tongue position, breathing, route, swallowing, and head and neck position in the pharyngeal airway [6]. This review aims to review the comprehensive role of CBCT in diagnostic, assessment, and analysis of OSA patients.

## 2. Materials and Methods

A comprehensive literature search was conducted using PubMed, Scopus, Web of Science and the Cochrane Library databases to identify relevant studies published between January 2015 and August 2025. The following keywords and Boolean operators were used: *“cone-beam computed tomography” OR “CBCT” AND “obstructive sleep apnea” AND “airway assessment” OR “airway analysis” OR “airway evaluation”*. Two reviewers independently screened titles and abstracts, followed by full-text assessment. Disagreements were resolved by consensus. Additional articles were identified by manually screening the references of selected studies.

Eligible studies included observational studies, diagnostic accuracy studies, interventional studies with pre- and post-treatment CBCT, and systematic reviews that reported on the diagnostic applications of CBCT for upper airway evaluation in OSA patients. The review included studies of all patients of any age with OSA with or without any intervention. Animal studies, case reports, debates between authors, conference abstracts, and papers not conducted using CBCT were excluded.

**Figure 1:**
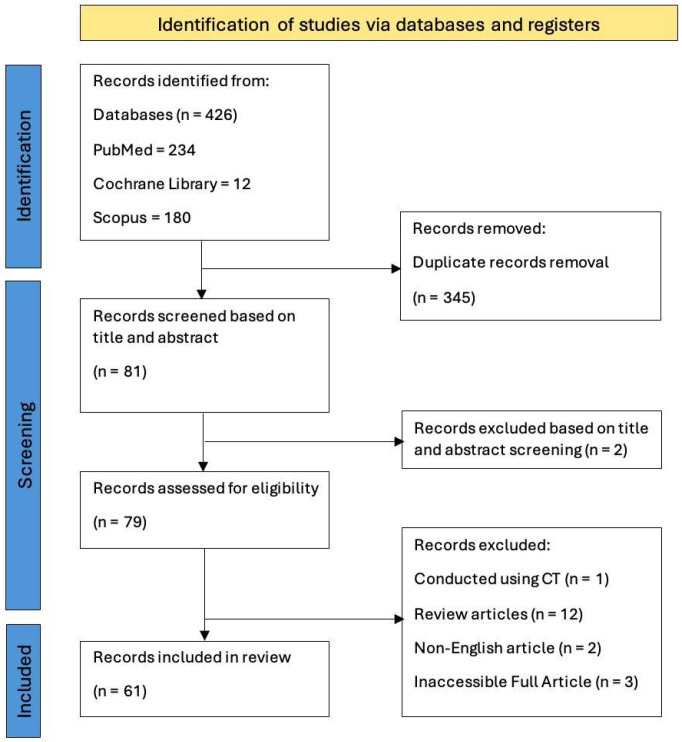
Flow diagram of the study selection process.

Among the 61 identified studies, a total of 2597 patients had undergone CBCT imaging. Airway assessment for OSA is the primary indication for CBCT recording in most papers. Secondary indications included evaluation for conventional orthognathic surgery, and cleft patients. A final number of 61 papers were included in this review (Figure 1).

A formal quantitative risk-of-bias tool was not applied because of the substantial heterogeneity in study designs, sample size, imaging protocols, acquisition parameters, outcome definitions, and reporting standards across the CBCT airway literature, which limits the validity of direct numerical scoring and cross-study comparison.

The review focused on the indications and patient position, as well as parameters, anatomical landmarks, and dimensions that make CBCT a reliable imaging technique to assist practitioners in managing patients with OSA. Heterogeneity including variability in airway boundary definitions, upright versus supine acquisition, static awake imaging, and device- or software-dependent segmentation was explicitly synthesized and critically discussed.

## 3. Anatomical Considerations in CBCT Airway Analysis: Landmarks, Boundaries, and Quantitative Parameters

To recognize the role and significance of CBCT in OSA, it is necessary to understand the complex upper airway anatomy. Generally, the airway can be divided into the upper and lower airway components. The upper airway is formed by the nasal cavities, pharynx, and larynx. The lower airway consists of the trachea, bronchi, and lungs. OSA is related mainly to the upper airway space where obstructions or constrictions can occur at any one or involving multiple levels. The upper airway is formed by the nasal cavity, pharynx, and larynx. It extends from the oral and nasal cavities up to the larynx, which functions as a gateway to the lower airway, beginning inferior to the vocal cords. The soft palate divides the nasopharynx and oropharynx while the tip of epiglottis divides the oropharynx and hypopharynx.

**Figure 2:**
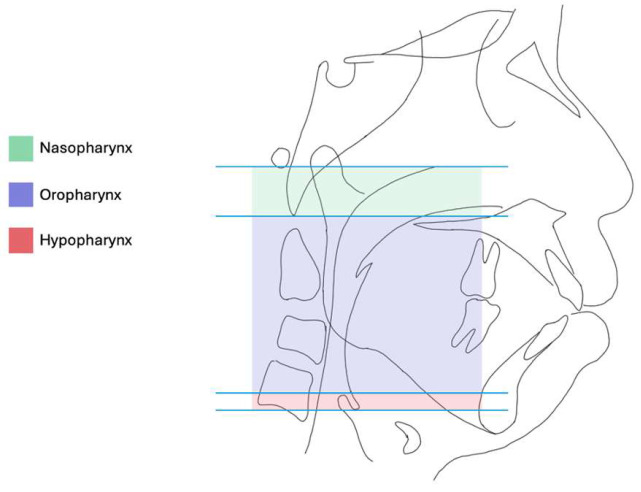
The upper airway is segmented into three parts: nasopharynx, oropharynx, and hypopharynx.

Variable terms have been used in the literature to describe the upper airway space as whole or as separate levels and subdivisions including superior/inferior upper airway, pharyngeal airway, naso-/oro-/hypopharynx, velopharynx, retropalatal space, retroglossal space, and others, reflecting the heterogeneity of nomenclature in airway research. Despite the variability, the anatomical division of nasopharynx, oropharynx, and oropharynx is broadly accepted and adopted for reporting and description in both radiological and surgical context (Figure 2). Clinically, the upper airway is often functionally subdivided into retropalatal and retroglossal segments, representing the two regions most frequently implicated in OSA-related collapse. Consistent with prior literature, the retropalatal level extends from the PNS to the uvular tip or inferior border of the soft palate, whereas the retroglossal level spans from the uvular tip to the base of the epiglottis [10,11].

### 3.1. Reference Landmarks and Its Clinical Relevance for Upper Airway Assessment in CBCT

In CBCT-based evaluation of the airway, the choice and reproducibility of anatomical landmarks are critical for ensuring valid, comparable measurements. Ideally, landmarks should be meaningful, easily identifiable across patients, and highly repeatable across observers. Commonly used hard- and soft tissue reference points include ANS, PNS, soft palate, uvula, epiglottis, cervical vertebrae (C1–C4), and the hyoid bone. Numerous studies have demonstrated high reliability, accuracy, and precision in identifying these landmarks on CBCT [12]. Yet, as highlighted by Yao et al. (2023) [13], landmark usage remains highly heterogenous, including 126 potential cephalometric landmarks, of which only six were specific airway points [14].

This diversity underscores a key challenge in airway research: different studies segment the “same” anatomical airway using different planes and reference systems, with cross-study comparisons. Table 1 summarizes commonly used craniofacial and pharyngeal landmarks relevant to CBCT airway assessment.

### 3.2. Anatomical Boundaries for Upper Airway Assessment in CBCT

**Figure 3:**
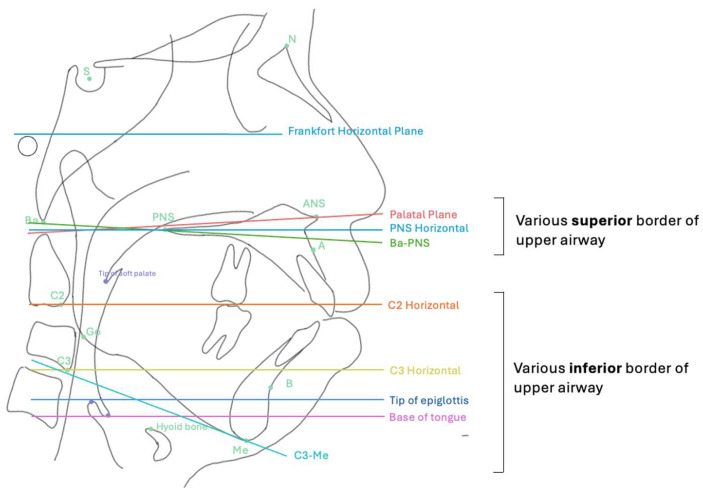
Illustration of the upper airway, showing the range of superior and inferior limits described in previous studies for airway measurement. Cephalometric landmarks: A: A Point; B: B Point; S: Sella; N: Nasion; Ba: Basion; Me: Menton; ANS: Anterior nasal spine; PNS: Posterior nasal spine; C2: Second cervical vertebra; C3: Third cervical vertebra.

A major source of variation in the CBCT airway literature concerns the definition of superior and inferior boundaries used during segmentation (Figure 3). No consensus currently exists on the optimal limits for airway assessment boundaries. Example, Ogawa et al. [15] measured airway volume between PNS and the anteroinferior point of C2, whereas Fonseca et al. [16] used a much longer segment extending from PNS to the mid-C4 level. Other studies have used the tip [11,17,18] or base of the epiglottis [11] as the inferior limit, particularly when focusing on the retroglossal airway. We summarized the boundaries of upper airway and segmentation in Table 2.

Conducting a systematic analysis, Palomo et al. [19] identified four commonly used upper limits and eight different lower limits that were commonly used in studies. The study demonstrated that volume and minimum axial area (MAA) measurements are highly correlated. This indicates that while absolute values differ when boundaries are shifted posteriorly or inferiorly, relative patient-to-patient differences and trends remain consistent, supporting the pragmatic use of standardized boundaries in future research. However, the choice of limits has practical consequences. Superior planes positioned too close to the nasal floor or posterior to septal fusion can cause software algorithms to misidentify the “vertex” of the segmented volume as the MAA. Conversely, inferior limits placed too high may artificially enlarge the measured MAA by excluding lower, narrower regions of the pharynx. This sensitivity highlights why clear, reproducible segmentation rules are important, especially when MAA is used as a clinical surrogate for airflow resistance.

Clinically, although absolute thresholds vary between studies, MAA values in the range of approximately 50–60 mm^2^ or below have been associated with increased collapsibility in several OSA-focused analyses. These values should be interpreted cautiously, given heterogeneity in imaging protocols and patient positioning, but they illustrate the potential relevance of MAA as a risk indicator in conjunction with clinical and polysomnographic findings.

## 4. Role of CBCT as a Diagnostic Adjunct in OSA Evaluation

CBCT can be utilized for various roles in the evaluation of OSA including as a diagnostic adjunct, preoperative surgical planning tool, and postoperative evaluation modality. Polysomnography may be the gold standard for OSA diagnosis; however, visual imaging is still required to provide information especially in localizing the site of obstruction.

### 4.1. Linear Dimensions

Airway analysis is traditionally performed two-dimensionally on lateral cephalogram or even lateral skull radiograph, involving mainly anteroposterior linear measurements. Using three-dimensional (3D) imaging such as CBCT, both anteroposterior (AP) and lateral (L) measurements can be determined as the shortest distance from one anatomical landmark to another in different levels of the airway. A study reported significant differences in both AP and L dimensions on the smallest axial slice in CBCT between the control group and the OSA group [20]. McNamara (1984) reported that a lower pharyngeal airway AP measurement of less than 10 mm indicates potential airway narrowing in OSA risk screening for [21]. It has been suggested that this cephalometric value may be translated to and applied as a reference threshold in CBCT-based studies. Studies have investigated the lateral width at the retropalatal and retroglossal levels and found that OSA patients may exhibit narrower airway transversely even when the AP dimensions show no significant differences [20]. A study found that an airway width of less than 17 mm increases the risk of OSA [22,23]. Lateral airway dimensions have been proven to be more reliable and consistent as a risk indicator for OSA compared to AP linear dimension which showed variable results across studies [2].

### 4.2. Cross-Sectional Area (CSA) or Minimum Axial Area (MAA)

The cross-sectional area (CSA) can be at any level or at specific levels of the upper airway, with two most popular sites being the retropalatal and retroglossal levels. The incorporation of software in airway analysis allows automatic computation of the CSA at every axial slice along the entire length of the pharyngeal airway space and detection of the slice with the smallest area, representing the MAA.

Studies have proven that CSA is significantly smaller in the OSA group versus the control group [24,25]. Fonseca et al. (2023) reported that an mCSA of less than 52 mm^2^ has a high risk of OSA [4]. In a latest systematic review by Kazmouz et al. (2025), the effect of CSA on OSA is reported to be variable, and only slightly more than half of the studies (57%) found the MAA to be significantly smaller in OSA subjects [11].

### 4.3. Total Airway Volume

The most commonly used outcome measurement is the total upper airway volume, which typically spans from the nasopharynx to the hypopharynx level. CBCT can determine the airway volume using a 3D programming software. This parameter offers a complete assessment of the airway lumen and detects volumetric measurement. Segmental volumes (retropalatal and retroglossal) provide spatially resolved assessment of potential obstruction sites. There is reported greater intra- and inter-observer reliability for airway volume compared to min. CSA [26]. The total airway volume of OSA subjects has been found to be significantly smaller [20,27]. Studies have found that OSA subjects have significantly longer airway [10,20,28].

### 4.4. Total Airway Length

The total airway length is another commonly assessed parameter. The airway length may be measured from the level of the palatal plane, the posterior nasal spine (PNS) to the anteroinferior of C2, or up until the lowest border of C4 distance [3,29]. Despite the variable anatomical landmarks proposed for airway length measurement, the PNS-C2 distance remains the most frequently used, as it is thought to more accurately reflect the length of soft tissue airway [3]. Airway length has been shown to have strong correlation with OSA, as explained through Poiseuille’s equation, which states that length influences resistance, with longer airways generally presenting with increased resistance. Studies have reported that airway length decreases after surgery in OSA patients [3,30]. Airway length is the only parameter in the measurement that decreases in number, compared to other measurements (e.g., airway volume, minimal cross-sectional area). Length metrics are less commonly used but can be informative in conjunction with volume and mCSA.

### 4.5. Airway Morphology

A normal airway usually displays as round- or square-shaped compared to the airways of OSA patients which are more elliptical [27,29]. This is because an elliptically shaped airway has a more natural tendency for collapse especially when the patient is in a supine position due to gravitation pull [31]. In addition, it is postulated that airway shape may influence the dynamics of airflow [11]. In contrast, other studies found no significant differences in the morphology of airway between the OSA and non-OSA group [10,32].

## 5. Role of CBCT in Craniofacial Hard and Soft Tissue Analysis in OSA

Besides allowing cross-sectional area, linear dimension, and 3D volumetric measurements, CBCT plays an indispensable role in airway assessment by providing structural and morphological information.

### 5.1. Role of CBCT in Hard Tissue Assessment and Its Effect on OSA

CBCT allows craniofacial skeletal analysis, including mandibular retrognathia or maxillary deficiency and hyoid bone positions. Craniofacial abnormalities or disharmony is a well-established predisposing factor in the pathogenesis of OSA. CBCT provides both two-dimensional and three-dimensional views of the hard and soft tissue anatomical details [33], which are often risk predictors of OSA.

#### 5.1.1. Cranial Base

Among the skeletal determinants, the cranial base angle—typically represented by the basion–sella–nasion (BaSN) or cranial base saddle angle—has been reported to be reduced in OSA patients, although the strength of correlation remains debatable. A narrower cranial base angle may lead to posterior displacement of the maxilla, reducing the anteroposterior (AP) dimension at the nasopharyngeal level and increasing airway resistance [11,34].

#### 5.1.2. Maxillary and Mandibular Skeletal Relationship

A recent systematic review and meta-analysis have found that retruded or short mandible and maxilla with low SNA and SNB angles are associated with OSA [11]. This forms the basis for MMA as one of the surgical interventions for OSA patients. Counterclockwise rotation of MMA with an increase in mandibular-plane angle, where the mandible autorotates forward to open up the pharyngeal airway space, has been found to be effective in the surgical treatment for patients with OSA [23,35,36]. Cephalometric studies also show that patients with OSA tend to exhibit increased anterior facial height and reduced posterior facial height (Ba–PNS distance) [14]. Among vertical parameters, lower facial height (ANS–Me) appears to have a stronger association with OSA risk than upper facial height (N-ANS) [11,29]. Larger facial height may correlate with an increased in total airway length, leading to increased airway resistance and predisposition to collapse. Collectively, these studies suggest that OSA risk is multifactorial, depending not on a single cephalometric parameter but on a combination of craniofacial dimensions affecting the upper airway segments.

#### 5.1.3. Hard Palate and Maxillary Arch Dimensions

Short transverse width and anteroposterior palatal length, represented by ANS-PNS distance are associated with increased likelihood of developing OSA [11,29,37]. Maxillary arch width may be measured as inter-first molar or first premolar distance [15]. The cause–effect relationship of a high-arched palate with OSA remains controversial. Compared to a square-arched palate, a tapered narrow palate may limit the space for the tongue in the oral cavity, displacing it backwards, leading to OSA [16,17]. On the contrary, some authors argue that chronic mouth breathing in OSA patients could be a secondary phenomenon that contributes to palatal constriction. Without the normal tongue position in place, there is a lack of lateral expansive forces on the palate, leading to transverse maxillary deficiency [18]. These bidirectional relationships suggest a complex interplay between airway obstruction, oral posture, and maxillary morphology.

#### 5.1.4. Hyoid Bone

The hyoid bone is a U-shaped bone that serves as an attachment or anchorage for several muscles including tongue musculature such as hyoglossus and suprahyoid (geniohyoid, mylohyoid, stylohyoid) and infrahyoid (sternohyoid and omohyoid) muscles. The hyoid bone is more inferiorly placed in patients with OSA [11,37,38,39]. Its position may influence the patency of the airway especially at the hypopharyngeal level. In OSA patients, poor muscle tone and chronic airway collapse may lead to the recruitment and overcompensation of infrahyoid muscles (functions to depress the hyoid bone) for pharyngeal airway stabilization. The hyoid bone also has a pulling effect on the genioglossus muscle, resulting in downward displacement of the tongue [40]. When added on with the presence of craniofacial deformities such as retrognathia and micrognathia, it may contribute to a further downward pull of the hyoid bone. The distance of the hyoid bone to the mandibular plane (MP) and to third cervical vertebra (C3) was also investigated in an airway assessment and the former has been shown to be an important marker in OSA prediction [14,41]. This enables the applicability of surgical procedures such as hyoid suspension or advancement to increase airway diameter. There is, however, a lack of strong evidence to support the positive correlation of hyoid bone to C3 distance [42,43,44]. Other cephalometric landmarks investigated in the literature include gonial angle, which showed no statistical significance [11].

### 5.2. Role of CBCT in Soft Tissue Assessment and Its Effect on OSA

Similarly, CBCT enables the evaluation of soft tissue structures that influence the airway, such as tongue size, tonsils, and adenoids [25,45,46,47]. Soft tissue points have been found to be highly reliable and reproducible in CBCT [48,49]. These landmarks also exhibited high inter-observer consistency and precision [12]. The tongue and soft palate are crucial soft tissue components which tonicity significantly affects the airway dimension.

#### 5.2.1. Soft Palate Morphology and Its Role in Airway Obstruction

Soft palate morphology is one of the most frequently evaluated soft tissue features in OSA. Soft palate parameters investigated in the literature include the length, width, thickness, and area. The palatal anteroposterior length, measured from the PNS to the tip of uvula, and thickness correlate with OSA severity [11,50]. The soft palate is dynamic and may regress and change over time with disease progression, becoming more flaccid and exacerbating the OSA symptoms. This pathophysiology underlies the role of uvulopalatopharyngoplasty (UPPP) and its variants as targeted surgical interventions for selected patients [29]. In addition, these soft palate parameters have been directly linked with obesity. Hence, weight reduction and bariatric surgeries have shown to positively improve the soft tissue obstructive effect on the airway [51].

#### 5.2.2. Tongue Morphology and Position

The tongue represents a major determinant of the oropharyngeal airway space. CBCT and MRI studies demonstrate that tongue size, volume, length, and height are significantly greater in OSA patients than in controls [11]. Tongue length is measured from the tip of tongue to the base of the epiglottis [15]. Excessive posterior tongue volume can encroach upon the airway, particularly in the presence of mandibular retrusion or hyoid inferiority. In contrast, adequate genioglossus tone during wakefulness maintains airway patency; the loss of this tone during sleep promotes posterior displacement and collapse.

## 6. Role of CBCT for Preoperative Assessment and Surgical Planning in OSA

In addition to its assessment and adjunct diagnostic role in OSA, CBCT is also significant in treatment planning, allowing simulation and providing pre-treatment visual representation. Identification and localization of a specific level of airway obstruction guide and aid in deciding the most appropriate therapeutic intervention tailored for the patient. CBCT has become integral in pre-surgical planning for OSA-related interventions, particularly MMA, orthognathic surgery, distraction osteogenesis, and nasal or palatal surgeries. Similarly, simulations in prediction of potential airway changes after orthodontic interventions such as rapid maxillary expansion, mandibular advancement device (MAD), or oral appliance therapy (OAT) may also be achieved [52]. A reduced MAA at the retroglossal level may suggest posterior displacement of the tongue and may benefit from MMA, genioplasty, or oropharyngeal soft tissue reduction. If lateral dimension narrowing at the retropalatal area is identified, it may reflect maxillary constriction or transverse deficiency, and the patient may benefit from surgical procedures such as surgically assisted maxillary expansion (SARPE) or mini-screw-assisted maxillary expansion (MARPE). Enlarged tongue or a narrower lateral transverse width might predict a lower CPAP compliance or success rate. Consequently, customization or titration of CPAP therapy according to airway characteristics on imaging can be performed [53].

In the context of orthognathic surgery, CBCT is used for virtual planning and surgical simulation besides predicting the amount of mandibular and maxillary movements and its impact on airway expansion [54,55]. CBCT enables precise quantification of skeletal movement and its effect on airway space. Three-dimensional reconstructions can be used to simulate forward and counterclockwise rotation of the maxillomandibular complex, visualizing predicted airway expansion [43,44]. Preoperative CBCT allows surgeons to evaluate posterior airway space at multiple levels—retropalatal, retroglossal, and hypopharyngeal—and anticipate the degree of volumetric improvement achievable post-surgery.

In addition, quantitative measurement of mandibular plane–hyoid distance (MP–H) and tongue volume allows selection of patients likely to benefit from targeted soft tissue advancement surgeries. For nasal surgeries such as septoplasty or inferior turbinate reduction, CBCT provides high-resolution visualization of nasal cavity and paranasal sinuses with accurate measurement of nasal airway volume. Similarly, in patients considered for uvulopalatopharyngoplasty (UPPP), CBCT can quantify palatal length and soft tissue thickness, guiding the extent of tissue resection and predicting postoperative airway change [47].

## 7. Role of CBCT for Postoperative and Longitudinal Outcome Evaluation in OSA Treatment

CBCT is valuable in quantifying postoperative airway change, allowing treatment efficacy evaluation after intervention. Sequential imaging allows for volumetric comparison of airway dimensions before and after interventions, for example, in airway dimension measurement and monitoring post MMA [56,57] or after continuous positive airway pressure (CPAP) [58]. In a meta-analysis of OSA surgical outcomes, patients undergoing MMA demonstrated an average 40–50% increase in oropharyngeal airway volume and a 60–80% reduction in AHI [51,52]. A retrospective study has reported a largely variable increment in airway volume and minimum axial area by 73.6 ± 74.75% and 113.5 ± 123.87%, respectively, after MMA [59]. Similarly, another study reported a mean increase of 2640 mm^3^ of the posterior airway space post MMA [60]. This is especially useful to motivate patients and predict long-term compliance. Postoperative CBCT has revealed sustained enlargement of the airway following MMA and genioglossus advancement even after soft tissue adaptation [61]. In addition to volumetric changes, CBCT can detect secondary skeletal remodeling and soft tissue adaptation, providing insight into relapse patterns and long-term stability. These data can be integrated into computational models for predicting patient-specific treatment durability.

## 8. Technical Considerations in CBCT-Based Airway Analysis

### 8.1. Workflow and Image Acquisition Protocols

CBCT-based airway analysis is highly sensitive to acquisition parameters, reconstruction algorithms, and segmentation methodology. Unlike conventional computed tomography, CBCT gray values are not standardized Hounsfield units (HUs) and vary according to scanner type, exposure settings, reconstruction filters, and proprietary software algorithms. First in the technical workflow would be image acquisition. CBCT imaging is usually acquired based on the machine’s specifications, with a recommended tube voltage of 90–120 kV, tube current of 5–10 mA, and voxel size of 0.3–0.4 mm, with a large field of view (FOV) of 6 × 13 cm or larger, and short scanning time. However, fixed threshold values reported in individual studies or embedded within specific software platforms cannot be directly extrapolated across devices and should be interpreted within their technical context.

CBCT imaging in the form of Digital Imaging and Communications in Medicine (DICOM) data may be extracted and imported into different software available in the market for analysis, which is further discussed in Section 8.3.

Before airway analysis, the CBCT image should be orientated, according to either the Frankfurt horizontal plane (FHP) or natural head position (NHP) and a defined vertical reference plane, usually the midsagittal plane. This is followed by definition of upper and lower airway boundaries where with the craniofacial complex and airway are captured preferably up to the fourth cervical vertebra.

Thirdly, threshold-based airway segmentation can be performed. Segmentation may be performed either by automation or manually. It is important to note that segmentation inaccuracy, artifacts (such as motion and scatter), and partial volume effect (PVE) in CBCT may affect volumetric measurement [62]. To reduce PVE and increase accuracy, fully automated airway segmentation using artificial intelligence has been suggested [60].

As such, this underscores a greater need for and importance of standardization and intra- and inter-observer reliability measurements in airway studies using CBCT.

Fonseca et al. (2023) [4] has recommended a standardized protocol for CBCT imaging in at risk patients using Planmeca ProMax 3D (Planmeca, Helsinki, Finland) and upper airway analysis with Romexis (version 5.1.O.R; Planmeca Oy, Helsinki, Finland) threshold set at 500, utilizing its in-built ‘Extract airways’ function tool for automatic calculation of airway dimensions including volume, area, and linear dimensions. It is crucial to take note of several technical factors that may influence the results of airway assessment, as summarized in Table 3.

### 8.2. Reference Planes and Patient Positioning

The Frankfurt horizontal plane (FHP), which connects the inferior orbital rim (orbitale) to the superior margin of the external auditory meatus, is the most commonly used reference plane in the literature. In upright imaging, the FHP is typically oriented parallel to the floor, whereas in supine imaging, it is aligned perpendicular to the floor [14,47]. Although no study has directly quantified the effect of FHP versus natural head position (NHP) on CBCT-derived airway dimensions, NHP has been recommended for preoperative orthognathic planning because of its reproducibility and physiological relevance [29]. In addition, CBCT reorientation protocols in the literature have used the palatal plane as the horizontal reference and either the midsagittal (glabella–crista galli–basion) or transporionic plane as the vertical reference axis [63].

Upright versus supine positioning differentially affects airway dimensions. Supine posture tends to reduce anteroposterior dimensions and MAA, particularly at the retroglossal level, while transverse dimensions and total airway volume appear more stable. Parameters most sensitive to positional change include minimum cross-sectional area and airway length [64,65,66]. This may be attributed to gravitational effects in the supine position. Interestingly, the impact of patient positioning on airway dimensions remains contradictory in the literature. Therefore, instead of assuming negligible influence, patient positioning should be considered when interpreting AP measurements, CSA, and segment-specific narrowing at the retropalatal and retroglossal levels. In addition, airway morphology is influenced by neuromuscular tone, which varies greatly during the sleep–wake cycle. During natural sleep, especially in the transition from wakefulness to non-REM and REM stages, pharyngeal dilator muscle activity diminishes, leading to increased collapsibility and dynamic narrowing that cannot be observed in the awake state [61]. Accordingly, CBCT acquired with the patient awake—whether upright or supine—only offers a partial view of airway behavior and does not replicate the physiological conditions under which OSA-related collapse usually occurs.

### 8.3. CBCT Machines and Software

Several studies have been performed to assess the reliability and accuracy of different software in airway analysis. In the literature, at least 20 software applications are used for image processing including Amira^®^ (Visage Imaging Inc., Carlsbad, CA, USA); 3Diagnosys^®^ (3diemme, Cantù, Italy); OnDemand3D^®^ (CyberMed, Seoul, Republic of Korea); Dolphin3D^®^ (Dolphin Imaging and Management Solutions, Chatsworth, CA, USA); MIMICS^®^ (Materialise NV, Leuven, Belgium); Romexis^®^ (Planmeca, Helsinki, Finland); ANSYS ICEM CFD 17.0 and ANSYS Fluent (ANSYS Inc., Canonsburg, PA, USA); ITK-SNAP; 3D Slicer; Dental Slice^®^; CS 3D Imaging Software; and OnDemand3D App. 17.0, ANSYS Fluent, Maxilim, InVivoDental, 3D Slicer, INTAGE, Volume Editor, ITK-Snap, Torrance, Vworks, 3dMDVultus, Invivo5, Analyze, Dental Slice^®^, CS 3D Imaging Software, and OnDemand3Dapp. A systematic review and meta-analysis by Gurgel et al. (2023) [2] revealed that the Dolphin software was the most commonly utilized software reported with moderate reliability. However, there is a paucity of studies that compare the identification and reliability of cephalometric anatomical landmarks using different CBCT machines. Studies have reported the use of the following tomographs, namely NewTom 3G; i-CAT^®^; PaX-Reve3D; Alphard VEGA; 3D Accuitomo; 3D eXam; PaX-Zenith3D; GALILEOS^®^; and CB MercuRay^®^, with NewTom 9000 being more commonly used in the oral and maxillofacial regions, but with limited applicability due to small FOV [48,67].

## 9. Limitations and Recommendations

### 9.1. Comparison of CBCT with Other Imaging Modalities

There are some limitations related to static CBCT imaging. CBCT provides a snapshot of the upper airway and cannot replace functional or dynamic assessments. It does not capture changes in neuromuscular tone, sleep-dependent airway behavior, or respiration-related alterations. Although CBCT is useful for anatomical characterization, its findings should be integrated with clinical, endoscopic, or sleep study data when evaluating functional obstruction in OSA.

CBCT represents a static, awake snapshot of the upper airway and does not capture sleep-related reductions in neuromuscular tone or dynamic collapse. Consequently, CBCT cannot substitute for polysomnography, drug-induced sleep endoscopy, or dynamic imaging modalities. Its findings should be interpreted as an anatomical predisposition rather than functional obstruction.

Other imaging modalities are available to accommodate the clinical evaluation process in the assessment and treatment planning of OSA patients, such as conventional computed tomography (CT) scan and magnetic resonance imaging (MRI). CT scan offers a key advantage in its ability to produce high-resolution images in multiple planes and sections with the capacity to generate numerous thin-slice sections for detailed anatomical evaluation. This makes CT scan particularly useful for visualizing complex structures in the craniofacial and airway morphology. However, the exposure of radiation is higher compared to CBCT.

MRI, on the other hand, is highly effective in establishing accurate soft tissue structure with high contrast resolution, enabling accurate assessment of the airway soft tissue morphology. Despite the advantage, the application is often limited due to its drawbacks, such as its prolonged image acquisition time, higher cost, and reduced accessibility in some clinical settings [29]. A form of MRI called dynamic MRI, allows evaluation of the airway in real-time while simulating natural sleeping positions. In contrast to conventional MRI which provides static anatomical images, dynamic MRI involves fast acquisition sequences such as Fast Low-Angle Shot (FLASH) or Ultrafast MRI for visualization and detection of dynamic airway collapse. Imaged acquisition is faster with lower acoustic nose level. A drawback is that patients may find it difficult to fall asleep in the scanner; however, a study by Volner [68] stated most participants (240 of 364) were able to sleep. Alternatively, sedation can be used to induce sleep during dynamic MRI scans, though it may alter the muscular tone when compared to natural sleep.

### 9.2. Integration with Computational Fluid Dynamics

Beyond static morphometry, CBCT serves as the anatomical foundation for computational fluid dynamics (CFD) analysis, a technique that simulates airflow patterns through the airway based on patient-specific geometry. Parameters such as airway resistance, pressure drop, and turbulent flow regions can be estimated, providing functional insight into airflow limitation. Although computationally intensive, this hybrid approach bridges the gap between anatomical imaging and physiological function, moving toward personalized surgical planning in OSA.

### 9.3. Incorporation of Artificial Intelligence (AI)

Automated airway segmentation, volume computation, and anomaly detection may be performed with high accuracy using deep learning algorithms of artificial intelligence.

In conclusion, CBCT is a widely accessible and clinically valuable adjunct for anatomical characterization of the upper airway in OSA, but it does not replace polysomnography nor capture dynamic airway behavior. In soft tissue delineation or functional collapse pattern and localization, MRI or drug-induced sleep endoscopy (DISE) may be preferable. When interpreted within its limitations and integrated with clinical and sleep data, CBCT remains a reliable and more readily available diagnostic imaging tool in OSA airway assessment.

## Figures and Tables

**Table 1:** Commonly used craniofacial hard- and soft tissue anatomical landmarks reported in the literature.

Structure	Anatomical Landmarks	Reported in OSA Association
Hard tissue		
Cranial base	BaSN angle	Reduced in OSA
Maxilla/Mandible	SNA, SNB ANS-Me, Ba-PNS	Retrusion linked to OSA Increased height, increased airway length
Hard palate	ANS-PNS Intermolar/premolar width	Narrow/tapered shows high risk of OSA
Hyoid bone	MP—Hyoid Hyoid—C3	Inferior positioning → high risk OSA
Soft tissue		
Soft palate	PNS—uvula	Correlates with OSA severity
Tongue	Tip–epiglottis, volume/dimensions	Larger size linked to OSA

**Table 2:** Core protocol for airway segmentation.

Reference Plane	
Frankfort Horizontal Plane (FHP) Natural Head Position (NHP)	
Level of Boundaries	Anatomical Landmarks
Superior border of nasopharynx	Sella—PNS Plane SS Plane S-Ba-PNS Plane Apex of nasopharynx Last slice before nasal septum fused with posterior
Inferior border of nasopharynx, or superior border of oropharynx	Palatal Plane (ANS—PNS) Horizontal Plane through PNS Plane connecting the odontoid apex to the PNS Ba-PNS Plane
Inferior border of oropharynx, or superior border of hypopharynx	Horizontal plane at the lowest anterior point of C2 Horizontal plane at the lowest anterior point of C3 Horizontal plane at the tip of the epiglottis Horizontal plane at the base of the epiglottis (vallecula) Plane connecting the lowest anterior point of C3 to the menton (Me) Plane connecting the highest anterior point of C4 to the menton (Me)
Inferior border of hypopharynx	C3—Menton C4—Hyoid bone Horizontal plane through the base of epiglottis

**Table 3:** Factors influencing airway analysis.

CBCT Machines and Image Acquisition Protocols
Reference planes
Position—supine and upright
Software for airway segmentation and analysis
Anatomical landmarks and boundaries

## Data Availability

No new data were created or analyzed in this study. Data sharing is not applicable to this article.
